# Inheritance patterns of the transcriptome in hybrid chickens and their parents revealed by expression analysis

**DOI:** 10.1038/s41598-019-42019-x

**Published:** 2019-04-08

**Authors:** Hongchang Gu, Xin Qi, Yaxiong Jia, Zebin Zhang, Changsheng Nie, Xinghua Li, Junying Li, Zhihua Jiang, Qiong Wang, Lujiang Qu

**Affiliations:** 1 Department of Animal Genetics and Breeding, National Engineering Laboratory for Animal Breeding, College of Animal Science and Technology, China Agricultural University, Beijing, China; 2 Institute of Animal Science, Chinese Academy of Agricultural Sciences, Beijing, China; 3 Division of Population Genetics, Department of Zoology, Stockholm University, Stockholm, Sweden; 4 Department of Animal Sciences, Center for Reproductive Biology, Veterinary and Biomedical Research Building, Washington State University, Pullman, United States; 5 Key Laboratory of Sustainable Development of Marine Fisheries, Ministry of Agriculture, Yellow Sea Fisheries Research Institute, Chinese Academy of Fishery Sciences, Qingdao, China

## Abstract

Although many phenotypic traits of chickens have been well documented, the genetic patterns of gene expression levels in chickens remain to be determined. In the present study, we crossed two chicken breeds, White Leghorn (WL) and Cornish (Cor), which have been selected for egg and meat production, respectively, for a few hundred years. We evaluated transcriptome abundance in the brain, muscle, and liver from the day-old progenies of pure-bred WL and Cor, and the hybrids of these two breeds, by RNA-Seq in order to determine the inheritance patterns of gene expression. Comparison among expression levels in the different groups revealed that most of the genes showed conserved expression patterns in all three examined tissues and that brain had the highest number of conserved genes, which indicates that conserved genes are predominantly important compared to others. On the basis of allelic expression analysis, in addition to the conserved genes, we identified the extensive presence of additive, dominant (Cor dominant and WL dominant), over-dominant, and under-dominant genes in all three tissues in hybrids. Our study is the first to provide an overview of inheritance patterns of the transcriptome in layers and broilers, and we also provide insights into the genetics of chickens at the gene expression level.

## Introduction

In recent animal genetic studies, a certain phenotype has been used as a starting point to study its mechanism, based on the laws of separation and combination proposed by Mendel^1^. The associated genes, genetic laws, and applications in breeding have become mainstream in genetic research. Similarly, for poultry, and particularly chickens, the genetic factors underlying many phenotypic traits have been studied in detail. For example, at the DNA level, phenotype traits of chicken, such as earlobe colour^2^ and polydactyly^3^, have been investigated using different approaches such as genome-wide association studies and re-sequencing analysis. Genes and their allelic relationships that affect eggshell^4,5^ and feather colour^6^ have also been characterised at the RNA level. In most cases, phenotypic differences between species are caused by genetic changes, these genetic differences are closely related to the expression and function of gene products^7^. We can thus determine such genetic differences according to differences in abundance at the transcriptome level. RNA-Seq technology has provided researchers with a powerful tool for the characterization and quantification of the transcriptome^8^. The abundance of target transcripts can be determined from an analysis of RNA-Seq data and is linearly related to the read count data obtained from RNA-Seq^9^.

In animals, hybridization events are widespread and may result in heterosis, phenotypic novelty, and certain changes in production performance in F_1_ hybrids^10^. These phenomena can be attributed to different magnitudes and directions of gene expression. Accordingly, the hybrid effect can be understood from a novel perspective by measuring the inheritance patterns of transcript abundance in hybrids compared with their parents. Conserved genes generally show kinship similarity in terms of inheritance patterns. According to the polygenic hypothesis, quantitative traits are determined by many genes with minor effect and the gene effects are additive^11^. However, at the molecular level, there has been little research data to verify or refute this assumption. Therefore, examining additive effects in genetic studies is important for gaining an understanding multi-gene effects and exploring heredity, and some studies have indicated that most key genes are characterised by additive expression^12^. Many plant-based studies have shown that dominance patterns are closely related to novel or the non-mid-parental phenotype of offspring, as even subtle changes can lead to significant phenotypic effects^13–16^. Moreover, mis-expressed (over- and under-dominant) genes contribute to heterosis and hybrid weakness in the hybridization process. Studying the extent of all inheritance patterns in hybrids can provide insights into the characteristics of genes in the hybridization between layer and broiler chickens. Although there have been many studies on inheritance pattern, most have focussed on plants and the model insect Drosophila. Furthermore, at present, there has been relatively little analysis of the effects of hybridization on gene expression in chickens and other commercially bred animals.

In this study, we examined the divergence in transcriptome expression of the hybrid progeny of broilers and layers, compared gene expression levels in the parents and their hybrids, and classified inheritance patterns according to a uniform standard. By altering gene expression during hybridization, different modes of gene action play different roles in the adaptation and evolution of species. Understanding the inheritance patterns of chicken transcriptome are crucial for finding key genes and understanding the molecular mechanisms of heterosis. By comparing with previous studies on plants or Drosophila, White Leghorn and Cornish are more able to accurately reflect the characteristics of hybridization occurs in domesticated animals.

## Materials and Methods

### Sample preparation

**Figure 1:**
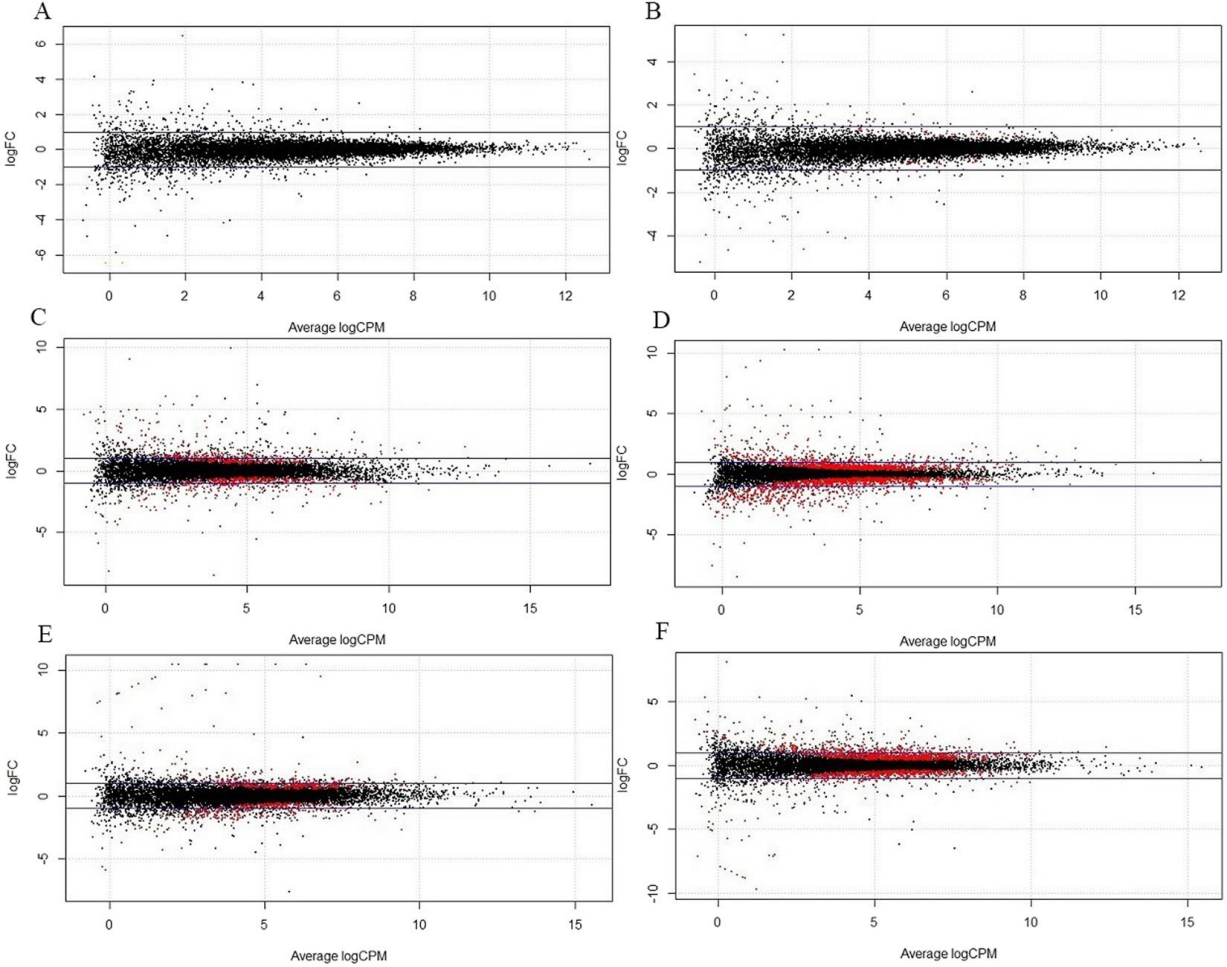
Samples and experiment design. CC and CW represent the offspring of Cornish (Cor) and White Leghorn (WL) respectively. LC represents the F_1_ hybrids of WL males and Cor females, whereas CL represents the F_1_ hybrids of Cor males and WL females. Samples of three tissues (brain, liver, and muscle) were collected from all chicks one day after hatching for transcriptome sequencing, and parental DNA extracted from blood samples was used for whole-genome re-sequencing.

We used White Leghorn (WL) and Cornish (Cor) as representative breeds of layers and broilers respectively, to obtain pure-bred and hybrid progeny (Fig. 1). Six offspring (three males and three females) were selected from the progeny of each mating, with the exception of the Cor male × WL female cross, for which only two female offspring (CL) were obtained. In order to ensure consistency in the number of samples and the sampling time, thereby minimizing differences due to distant kinship between Cor males and females, we used two groups of three males and three females obtained from Cor male × Cor female and WL male × WL female matings, designated CC and CW, respectively, to represent the parents of the hybrid offspring.

For samples of three tissues (brain, liver, and muscle) collected from 23 one-day-old chicks, we extracted total RNA using Trizol reagent. And the total RNA was sequenced using 100-bp paired-end reads and 300-bp insert size. Finally, we obtained a total of 246.3 Gb of RNA-Seq data, corresponding to an average of 3.6 million mappable reads per sample.

All experiments were approved by the Animal Care and Use Committee of China Agricultural University (Approval ID: XXCB-20090209). All animals were fed and handled according to the regulations and guidelines established by this committee, and all efforts were made to minimize suffering and protect animal welfare.

### Parental genome reconstruction

To ensure accuracy of the RNA-Seq data mapping, it was initially necessary to reconstruct the four parental genomes instead of using the reference genome of chicken. We accordingly mapped re-sequencing parental data to the reference genome of chicken (Gallus_gallus-4.0, http://hgdownload.soe.ucsc.edu/goldenPath/galGal4/bigZips/) using the Burrows Wheeler Aligner (BWA)^17^. We then used Picard tools (http://broadinstitute.github.io/picard/) to sort the resulting bam file according to the order of chromosome coordinates, and Picard MarkDuplicates was used to remove duplicate reads. Finally, the Genome Analysis Toolkit (GAKT)^18^ was used to filter the original results and then complete SNP calling. If a sufficient number of reads appeared as variant types, the mutation site could be used to replace the corresponding site in the reference genome, and this operation was based on output results from Vcftools^19^. We then filtered SNPs in the two parental breeds, and SNPs supported by >10 reads in each parental breed were retained.

However, when the SNP sites were located on the Z chromosome, the threshold was lowered to >4 reads. These thresholds were set in order to eliminate potential sources of bias^20,21^.

### RNA-Seq data analysis

We aligned RNA-Seq data of pure-bred Cornish (CC), White Leghorn (CW) offspring to the chicken reference genome (Gallus_gallus-4.0) by using STAR^22^. The data for hybrid offspring (CL, LC) were mapped to the parental genomes that had been similarly reconstructed using STAR. Samtools^23^ was used to remove duplicate reads so as to eliminate potential deviations^24^. After mapping, we were able to obtain the resulting bam files. In order to call SNPs, these bam files were converted to vcf files using the mpileup function of Samtools. For CC and CW, featureCounts^25^ was used to estimate transcript abundance. For CL and LC, we used the asSeq^26^. package of R (v 3.4.2) to process these data, and finally obtained the total read counts (TReC) and allele-specific expression (ASE) counts of hybrids together.

### Normalization and filtering

In many similar studies, a total-count scaling method of normalization has been applied to standardise the total number of reads between lanes, such that the average expression level across all genes is the same in each library^27,28^. In the present study, we used a similar normalization method to reduce the bias in further steps. The mapped reads of each gene in three repetitions were initially averaged, and total expression was normalised by dividing the number of mapped reads at each gene by the total number of mapped reads for the entire genome, and multiplying by 100 to yield a percentage expression value. This step eliminated the expression divergence due solely to technical differences in sequencing depth between libraries and ensured the accuracy and credibility of inheritance classifications.

For all samples and all tissues, we filtered out genes for which the expression level was zero. These genes would not be expressed under any condition. In addition, dosage compensation in chicken is incomplete^29,30^, and thus the average expression of males (ZZ) is higher than that in females (ZW). Accordingly, to eliminate the dosage compensation effect due to gender, we also excluded genes on the Z chromosome. All genes satisfying the established thresholds were retained for further analysis.

### Inheritance classifications

The samples were divided into four groups, designated as the Male Cross (MC: WL♂, Cor♂, CL♂), Female Cross (FC: WL♀, Cor♀, CL♀), Male Reciprocal Cross (MR: WL♂, Cor♂, LC♂), and Female Reciprocal Cross (RF: WL♀, Cor♀, LC♀), in order to investigate differences in the effect between groups.

**Figure 2:**
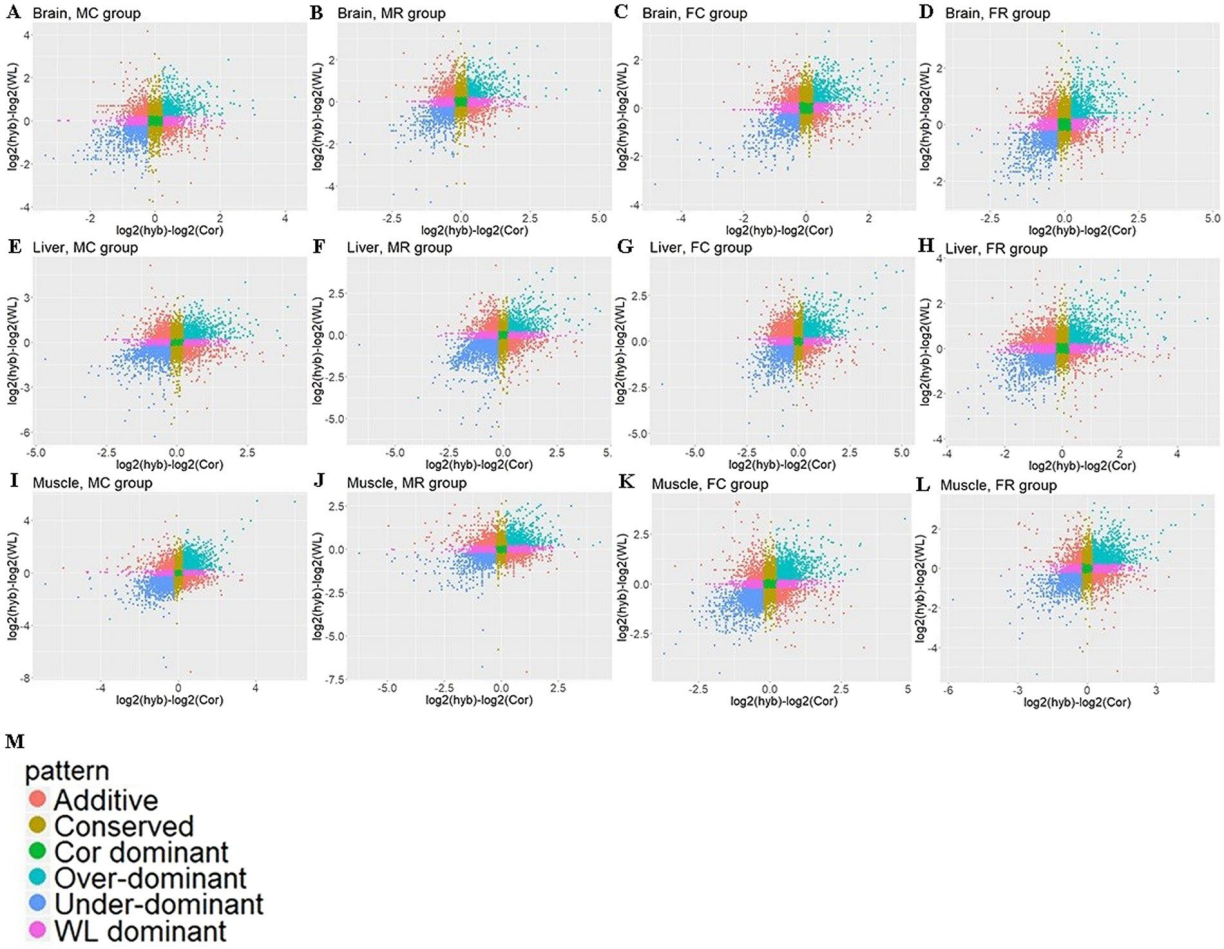
Hypothetical patterns of gene expression in Cornish (Cor), White Leghorn (WL), and F_1_ hybrids.

R (v 3.4.2) was used to sort genes in terms of differences in the expression levels between each parent and their hybrid. We converted the expression data of parents and hybrids into a log-transformed percentage, thereby enabling directions and fold changes to be viewed directly^31^. A threshold of 1.25-fold was set so that we could determine if there were significant differences in expression level^32^. Genes for which the expression differed by amounts less than this threshold were considered to have similar expression, and these genes were regarded as being conserved. In contrast, genes for which the expression differed by amounts greater than 1.25-fold were accordingly designated as non-conserved. Non-conserved genes were further divided into additive, dominant, under-dominant, and over-dominant. In this study, dominant genes were divided into two types, designated Cor dominant and WL dominant, for the purpose of accurate statistical analysis (Fig. 2).

### Differentially expressed gene functional annotation

We used the Functional Annotation tools of DAVID Bioinformatics Resources (6.8)^33^ to annotate the differentially expressed (DE) genes in the brain, liver, and muscle. Using this approach, we anticipated discovering certain gene functions related to the traits of broilers and layers.

## Results

### Sequencing and mapping

The genomes of a total of 23 individuals were sequenced using cDNA libraries. Reads obtained from Illumina runs were initially trimmed in order to remove low-quality reads. After trimming, we obtained an average 22.8, 17.8, and 21.3 million mappable reads per individual in the brain, liver and muscle, respectively.

We re-sequenced the parental genomes and then reconstructed the four parental genomes as substitutes for the reference genome of chicken. These parental genomes were used for next reads mapping and SNP calling. The SNPs were filtered and only those SNPs supported by >10 reads were used to assess ASE. After filtering, we obtained reads for 988 autosomal genes covered by 2564 SNPs and 81 Z-linked genes covered by 312 SNPs in the brain; 679 autosomal genes covered by 1,758 SNPs and 60 Z-linked genes covered by 235 SNPs in the liver; and 961 autosomal genes covered by 2,677 SNPs and 76 Z-linked genes covered by 248 SNPs in the muscle.

### Divergence in expression between layers and broilers

**Figure 3:**
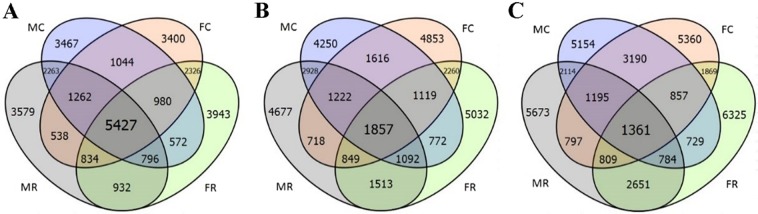
MA plot summarizing the expression divergence between parental layers and broilers of (**A**) male brain, and (**B**) female brain, and (**C**)male liver, and (**D**) female liver, and (**E**) male muscle, and (**F)** female muscle. LogFC indicates the fold change using logarithmic-transformed data. A positive logFC indicates that the layer parent has higher expression than the broiler parent, whereas negative values show that the broiler parent has higher expression than the layer parent. LogCPM represents the logarithmic foldness in counts per million. The blue horizontal lines indicate two-fold changes in expression, and red dots represent genes significantly up- or down-regulated.

We compared gene expression levels between White Leghorn and Cornish birds using edgeR analysis^34^, and the results were used to indicate the expression divergence between broilers and layers. Finally, we obtained the results of differential expression analysis for each of the three examined tissues (Fig. 3). The majority of DE genes had a less than two-fold difference in expression, indicating that the parental expression divergence is subtle. After filtering, we obtained 24267 genes detected in the brain tissue of both males and females, with the number of up-regulated and down-regulated genes being 104 (0.43%) and 91 (0.37%), respectively (FDR < 0.05). The number of genes detected in the liver and muscle, were 20943 and 22705, with 1502 (7.17%) up-regulated and 1704 (8.14%) down-regulated genes in the liver, and 1505 (6.63%) up-regulated and 1118 (4.92%) down-regulated genes in the muscle (FDR < 0.05). These results indicated that there is generally no clear expression divergence in the brain between layers and broilers. In contrast, we detected a significant divergence of expression in the muscle and liver, and these patterns can also be seen by the degree of dispersion in the expression divergence plot (Fig. 3).

### Transcriptome inheritance pattern classifications

After normalization and filtering the data, we obtained 15810, 14864, and 15390 genes that satisfied the filtering criteria for the brain, liver, and muscle. It was possible that the expression level of a certain gene is zero for all individuals in a group, in which case a classification of “0” was assigned in the output results. Because we removed these non-statistical outputs, the number of genes in the same tissue in different groups is slightly different in our final classification statistics.

**Figure 4:**
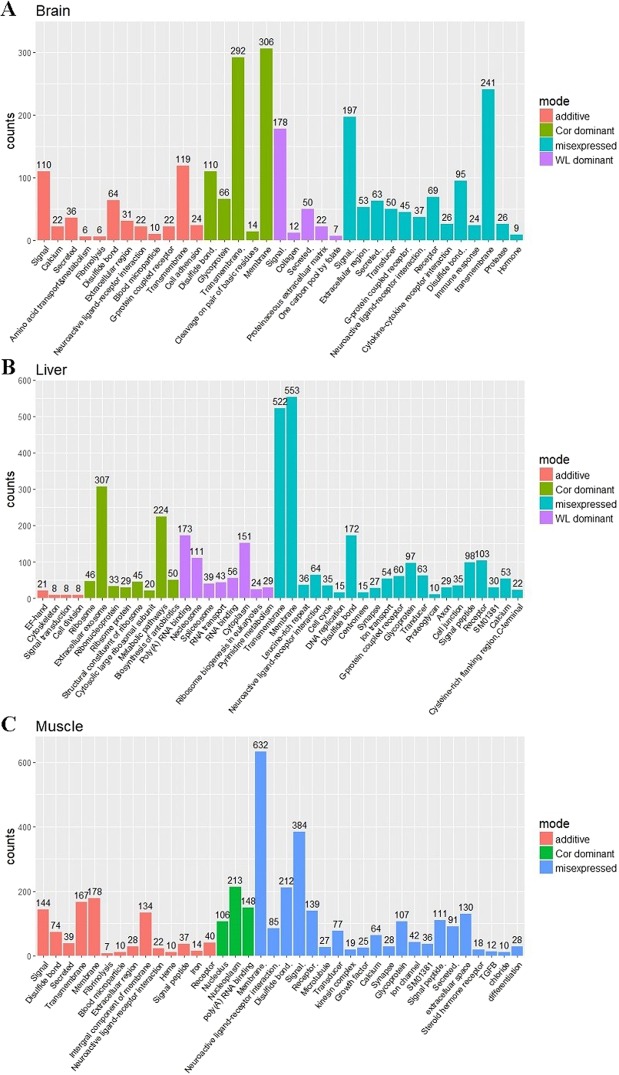
Scatterplots representing different inheritance patterns. The plots compare differences in the expression of genes between the F_1_ hybrid and those of the parental breeds for (**A–D**) four groups (MC, MR, FC, FR) in the brain, and (**E–H**) four groups in liver, and (**I–L**) four groups in the muscle. (**M**) The different coloured dots represent the different inheritance categories.

We used Scatterplots to visually represent relationships between the expression levels of F_1_ hybrids and their two parents (Fig. 4). Additive indicates the genes for which expression in the hybrids was between the expression levels of the two parents. Genes were classified as Cor dominant or WL dominant when the expression level in hybrids was only similar to that of corresponding parent. Genes for which the expression in hybrids was higher or lower than that in both two parents were regarded as over-dominant and under-dominant, respectively. On the basis of the statistical results (Table 1), conserved genes accounted for the largest proportion of those detected. This highly conserved pattern was particularly apparent in the brain tissue, in which more than half of all genes, 8754 (57.2%), 8688 (56.5%), 8389 (54.7%), and 7733 (50.8%), showed conserved expression in groups MC, FC, MR, and FR, respectively. The proportion of additive genes was comparatively small, accounting for 5.3%, 9.6%, and 7.3% of the total in the brain, liver and muscle, after being averaged among the four groups. We detected a significant difference in the number of mis-expressed (over- and under-dominant) genes among the three tissues (Kruskal–Wallis test, P-value < 0.05). Dominant genes showed more Cor-like expression than WL-like expression in the brain tissue (Mann–Whitney test, P-value < 0.05), whereas there was no difference between the number of WL dominant and Cor dominant genes in the liver [Mann–Whitney test, P-value = 0.88 (>0.05)]. Interestingly, in the muscle, genes showed more Cor-like expression than WL-like expression in the MC and FC groups (cross groups), but the opposite pattern in the MR and FR groups (reciprocal cross groups).

**Table 1:** Statistical result of inheritance classification in the brain, liver, and muscle.

Tissue	Group	Inheritance classification					
		Conserved	Additive	Cor dominant	WL dominant	Over- dominant	Under- dominant
Brain	MC	8754	847	2283	1497	1026	910
	FC	8688	822	2341	1461	1110	946
	MR	8389	882	2033	1969	1091	961
	FR	7733	715	2641	1470	1379	1286
Liver	MC	4317	1306	3083	2504	1720	1230
	FC	4195	1508	2795	2757	1615	1261
	MR	3883	1292	2477	3069	2318	1146
	FR	4393	1305	2352	2651	1632	1546
Muscle	MC	3900	961	3926	1775	2264	1946
	FC	4015	1066	3626	1946	2211	1889
	MR	5185	1169	1742	4136	979	1590
	FR	3772	1090	2864	2953	1408	2609

**Figure 5:**
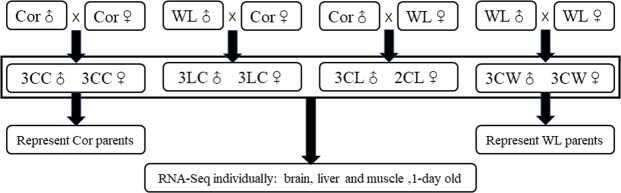
Four-way Venn diagram illustrating statistical results of inheritance modes in four groups (MC, MR, FC, and FR) correspond to (**A**) brain, and (**B**) liver, and (**C**) muscle. The number in each colour block indicates the number of genes for different inheritance patterns in single or multiple groups.

In the brain, there were 5426 genes showed the same inheritance mode in all four groups, among which 5138 genes (94.7%) were conserved. Similarly, there were 1851 and 1361 genes that showed the same mode of inheritance in the liver and muscle, with 1252 (67.6%) and 1054 (77.4%) genes being conserved (Fig. 5). The numbers of these genes in the liver and muscle tissues were clearly lower than those in the brain tissue. These results also indicated that the majority of genes were consistent expression among the four groups were conserved, and this pattern was more prominent for genes in the brain.

### ASE analysis of dominant

In order to assess ASE, the RNA-Seq data of progeny were aligned to the genomes of the two parents, and a threshold was used to filter unqualified SNPs (normalised reads <10). We performed the same normalization and filtering procedures for the ASE data as described above.

**Figure 6:**
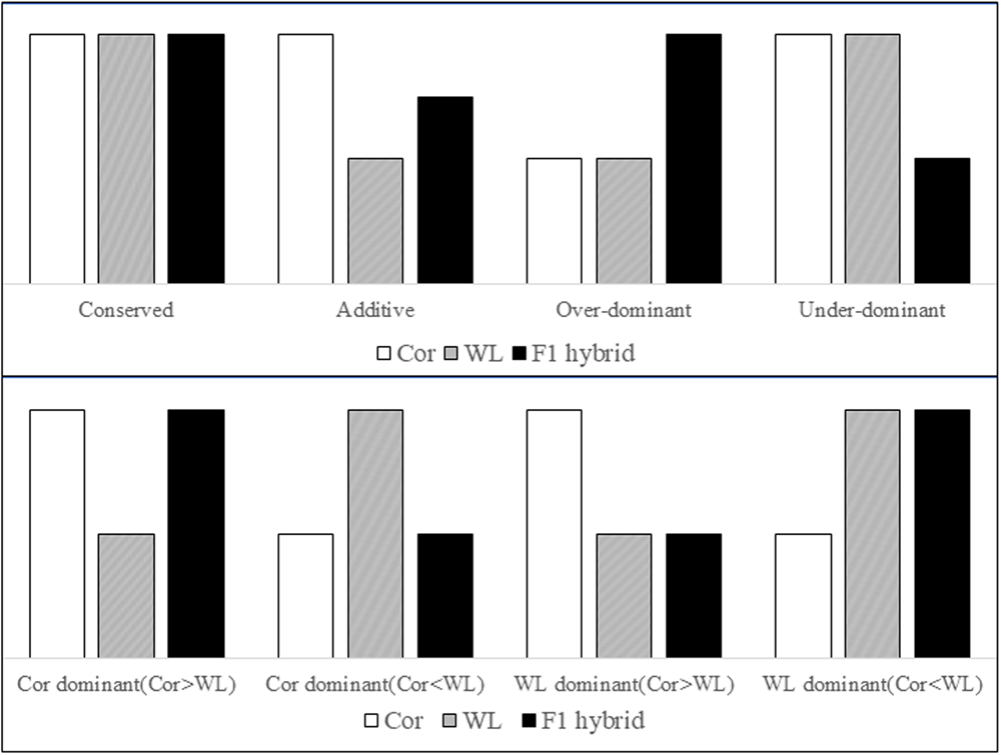
Correlation of the expression of Cor and WL in parents and two alleles of hybrid for dominant inheritance. The dominant pattern was divided into (**A**) Cor > WL and (**B**) Cor < WL according to expression of genes. The X axis shows the expression of pure-bred Cor or Cor allele in hybrids. The Y axis shows the expression of pure-bred WL or WL allele in hybrids.

To determine the relationship between the expression pattern of specific-expression alleles and the parental expression of dominant genes, we applied a test that compared the ratio of expression between parental breeds (Cor/WL) and two alleles of F_1_ hybrids in the three examined tissues of all groups, using log-transformed data. According to the expression level of the parental breeds, dominant was divided into two patterns: Cor > WL and Cor < WL (Fig. 6). This step could prevent masking of the real expression pattern because of neutralization in these two cases. There was a significant correlation between the ratio of Cor/WL alleles in F_1_ hybrids and their parental expression (P-value < 0.05, Pearson’s test, Supplementary Table S1). Furthermore, in the two cases of Cor > WL and Cor < WL, the slopes of the regression lines were close to 1 in the offspring and were 0.9874 (Cor > WL) and 1.0971 (Cor < WL) for the two parental patterns, whereas intercepts were approximately equal to 0 in the offspring and 0.1078 (Cor > WL) and 0.0233 (Cor < WL) for the two parental patterns (Fig. 6). These results indicated that although the ratio of the two alleles had a tendency similar to the ratio of parental expression, this tendency became inconspicuous. Moreover, on the basis of these results, it was evident that the two alleles had a similar level of expression.

### Gene function annotation

**Figure 7:**
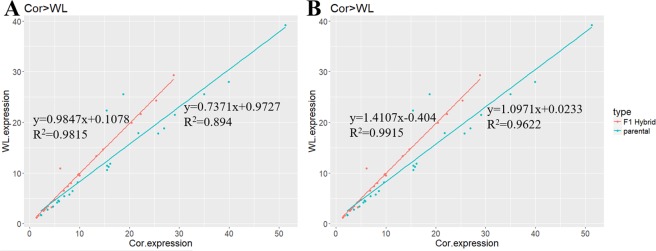
The significantly enriched biological functions of DE genes in (**A**) the brain, and (**B**) muscle, and (**C**) liver.

In order to ensure accuracy prior to the function annotation of DE genes, if any of these genes showed the same mode in two or more groups in a tissue, these genes would be considered as showing that inheritance mode in the corresponding tissue. DAVID was used to annotate genes of different modes in the three tissues, and we determined the functions of genes with significant enrichment by filtering (FDR < 0.05, Fig. 7).

After classification using the aforementioned step, we found that 859, 1673, 1446, and 1992 genes showed additive, Cor dominant, WL dominant, and mis-expressed expression in the brain, whereas the corresponding numbers in the liver and muscle were 1468, 2931, 2845, and 3038, and 1042, 3138, 2675, and 3421. As shown in Fig. 7, the function of significantly enrichment genes from left to right is arranged in ascending order of FDR value (significance of enrichment is descending) in each mode.

Among the annotation results, we found that more DE genes were enriched in the functions of membrane, transmembrane, and signal than any other biological functions. Furthermore, we found that biological functions were more significantly enriched (FDR < 0.05) in mis-expressed genes than in the other modes. However, for WL dominant expression in the muscle, no DE genes were significantly enriched, and the genes were related to a variety of diverse biological functions.

## Discussion

More than 90% of genes in the brain and muscle were retained following the edgeR filtration process, whereas the corresponding value for genes in the liver was 84.2%. After filtering, genes were used to identify DE genes in the parental breeds. We accordingly, observed very little significant divergence in gene expression, particularly in the brain. Divergence was found to affect 15.3% of genes in the liver and 11.6% of genes in the muscle. In the brain, only 0.8% of genes showed significant differential expression. And this inconspicuous expression divergence was also reflected in the research of Leghorns and Fayoumi chickens, which found 304 DE genes in the brain and 579 DE genes in the liver^35^. These proportions of gene expression are considerably lower than those which have previously been reported in *Drosophila*^7^ (78%) and *Cirsium* from natural populations^31^ (51%). The fact that a majority of DE genes had fold-changes of less than two indicates the existence of many subtle differences between populations. Collectively, these results indicate that a few decades of high-intensity selection for layers and broilers has not resulted in any obvious differentiation between the Cornish and White Leghorn breeds at the genetic level, and that this continuous selection of specific phenotypes and traits appears to involve fewer genes in the brain compared with genes in the liver and muscle.

This study revealed all gene action patterns in hybridization process, including conserved in which genes in F1 hybrids were not significantly different with both parents and other modes called nonconserved. Clearly, the latter is more meaningful to research because change in genetic levels may give rise to phenotypic or trait change. Previous studies on plants had suggested that both additive and non-additive effects are associated with heterosis at the molecular level^36^. Furthermore, recent research on gene expression patterns has shown that non-additive patterns are prevalent in inbred lines or different natural populations, and that they may play a certain role in heterosis^37^. In our study, conserved genes were most prevalent in the three tissues of all groups. The finding that more than half of the genes in the brains of the two parental chicken breeds showed conserved expression not only indicates that the brain of these two breeds have not diverged significantly, but also that genes in the brain have remained highly conserved in the process of hybridization. Indeed, this highly conserved pattern has been widely observed in other studies^10,38^. A majority (88% in the brain, 86% in the liver and 90% in the muscle) of DE genes showed non-additive (i.e., dominant, over-dominant, and under-dominant) patterns, which does not support multi-gene hypotheses because of the rare additive pattern. For the dominant inheritance mode, there were more genes (59%) that tended to be Cor dominant in the brain. Interestingly, dominant genes in the muscle showed clear gender divergence. In most cases (67%), dominant genes had a Cor-like expression pattern in male groups (MC, MR), whereas in contrast, female groups (FC, FR) showed high WL dominance pattern (61%). For mis-expressed genes, more showed an over-dominant rather than an under-dominant pattern, indicating that hybridization is often associated with the hybrid vigour of offspring instead of hybrid weakness, although some studies on interspecific hybrids have observed that the frequency of under-dominant is significantly higher than that of over-dominant. In order to distinguish whether there was a significant divergence between expression levels, we adopted a threshold of 1.25-fold to classify inheritance categories. However, the threshold determination criteria were flexible and we could also use the results of significance (binomial) tests in this study, whereby an FDR value greater than 0.5% signifies a similar expression. Although different threshold criteria may lead to more stringent or more lenient filter results, this would not have a substantial impact on our statistical analyses.

The expression of genes classified dominant was similar in hybrids and both of the two parents. We are not aware of any intrinsic regulatory mechanisms whereby the expression of dominant genes of F_1_ hybrids is similar to that of one parent and significantly different from that of the other parent. However, quantifying the direction and magnitude of ASE divergence is an effective approach for determining this type of mechanism. On the basis of our results for ASE of the dominant mode, a relative expression relationship of parental alleles also exists between two parental alleles in the hybrids. However, it should be noted that Cor/WL in hybrids was approximately equal to 1 (0.9847 in Cor > WL group, 1.0971 in Cor < WL groups), For dominant expression, the two alleles showed equal expression rather than a higher expression of one allele relative to that of the other allele. The ASE of heterozygotes is governed at the level of transcription by interactions between *cis*- and *trans*-acting regulatory elements^39^. *cis*-regulatory sequences have allele-specific effects on the expression of neighbouring genes, whereas *trans*-regulatory factors affect the expression of both alleles in a diploid hybrid cell. In studies on other species, certain associations between *cis*-regulation, parental expression differences, and inheritance patterns have also been found^40–44^. And recent study in chickens found that trans-regulation is the main cause of differential gene expression and thus contributes to heterosis effect in the F1 hybrids^35^.

Given, the low genetic divergence between the two breeds of chicken examined in the present study and the fact that we used day-old chicks that may have been too immature to show certain characteristics of layers and broilers, it might not be surprising that we were unable to detect large numbers of DE genes significantly enriched for particular functions. Indeed, most genes corresponded to a variety of diverse biological functions rather than being mostly related to biological functions associated with the production of eggs and meat. However, we still identified metabolic pathways, pyrimidine metabolism, and ion transport, these annotated results are consistent with the material metabolism function of the liver. Growth factor, TGFB, and calcium were significantly enriched when we annotated the DE genes in the muscle, and growth factors can promote cell proliferation, while TGFB can promote cell differentiation and embryo development, these substances play an important role in the growth and development of muscles. Interestingly, these gene functions are not enriched in Cor dominant, but are enriched in mis-expressed.

Different inheritance modes can provide insight into the genetic mechanisms of hybridization in different aspects. The study of these modes and expression divergence in parents also reflects the extent to which artificial selection can alter the genetic background of layer and broiler chickens, and this extent of change can be measured by comparison with studies using naturally selected populations. Because of the technical and software limitations of SNP calling and the wide distribution of SNPs in the genome^29^, it was not possible to detect all SNPs in layer and broiler parents. Therefore, we could not obtain or measure the ASE data in genes corresponding to undetected SNPs. However, the informative SNPs represented only a small proportion of the total SNPs detected. Furthermore, we also used stringent filtering criteria, and the ASE results were not affected and the credibility was still high. In the future, technology based on a more advanced platform, greater optimization of analysis methods, and a broader range of samples and developmental stages for differential expression analysis will further promote our understanding of the characteristics of each inheritance pattern and enable us to predict genes or traits that show heterosis and hybrid weakness.

## Supplementary information

Supplementary Information

## Data Availability

The datasets generated during and/or analysed during the current study are available from the corresponding author on reasonable request.
