# Publisher Correction: Inheritance patterns of the transcriptome in hybrid chickens and their parents revealed by expression analysis

**DOI:** 10.1038/s41598-020-63873-0

**Published:** 2020-04-17

**Authors:** Hongchang Gu, Xin Qi, Yaxiong Jia, Zebin Zhang, Changsheng Nie, Xinghua Li, Junying Li, Zhihua Jiang, Qiong Wang, Lujiang Qu

**Affiliations:** 1 Department of Animal Genetics and Breeding, National Engineering Laboratory for Animal Breeding, College of Animal Science and Technology, China Agricultural University, Beijing, China; 2 Institute of Animal Science, Chinese Academy of Agricultural Sciences, Beijing, China; 3 Division of Population Genetics, Department of Zoology, Stockholm University, Stockholm, Sweden; 4 Department of Animal Sciences, Center for Reproductive Biology, Veterinary and Biomedical Research Building, Washington State University, Pullman, United States; 5 Key Laboratory of Sustainable Development of Marine Fisheries, Ministry of Agriculture, Yellow Sea Fisheries Research Institute, Chinese Academy of Fishery Sciences, Qingdao, China

Correction to: *Scientific Reports* 10.1038/s41598-019-42019-x, published online 08 April 2019

This Article contains an error in the order of the Figures. Figures 1, 2, 3, 4, 5, 6, 7 are published as Figures 5, 6, 1, 2, 3, 7, 4 respectively. The Figure legends are correct.

In addition, there is an error in Figure 6B, where “Cor>WL” should read “Cor<WL”.

The correct Figures appear below.

**Figure 1:**
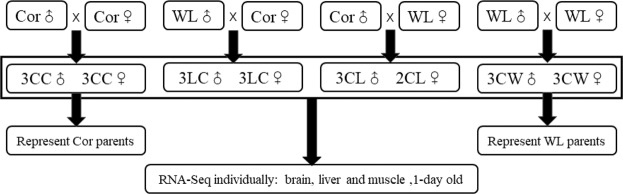
.

**Figure 2:**
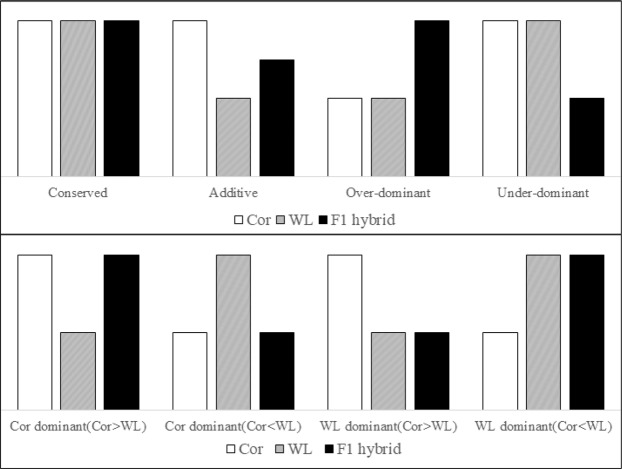
.

**Figure 3:**
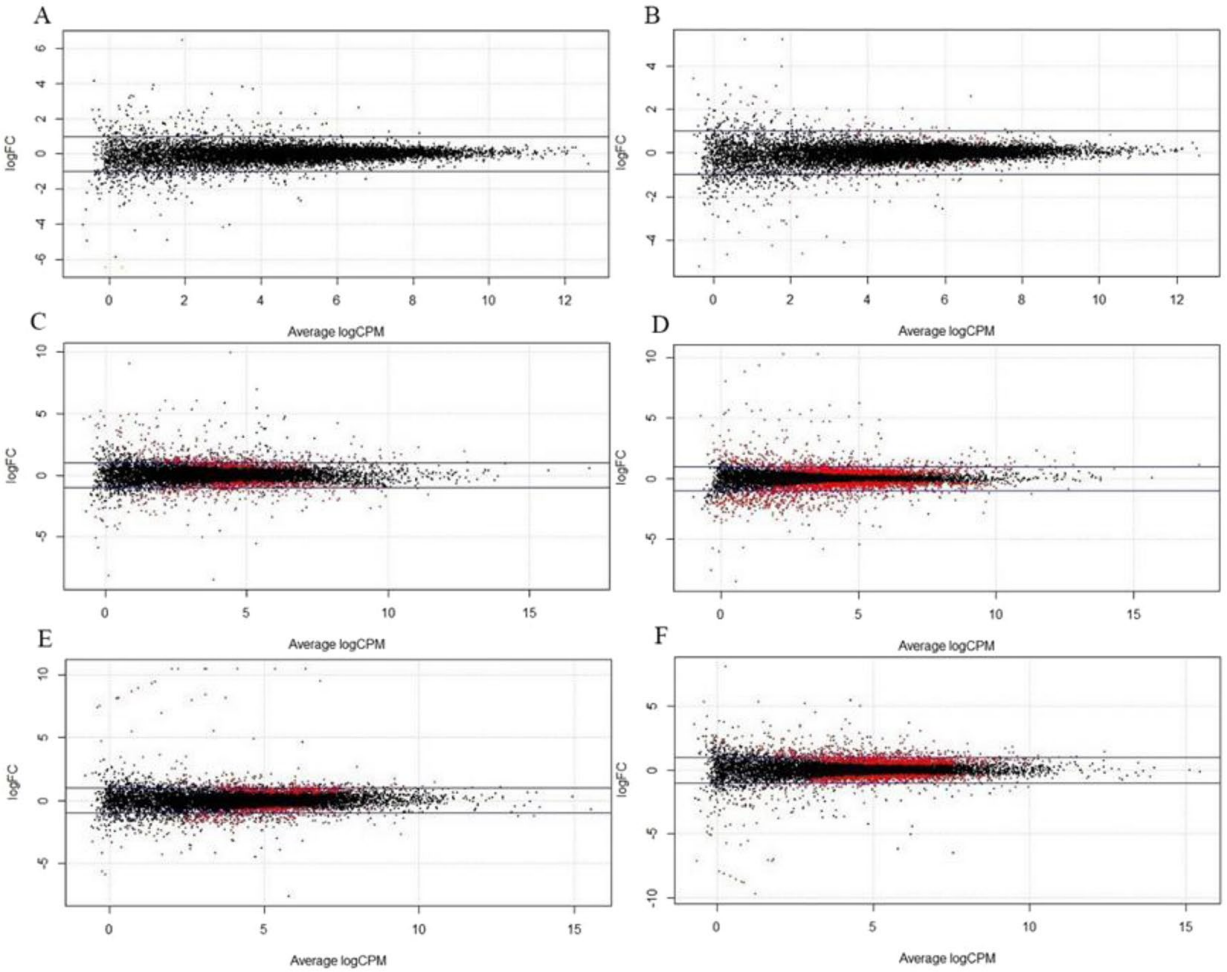
.

**Figure 4:**
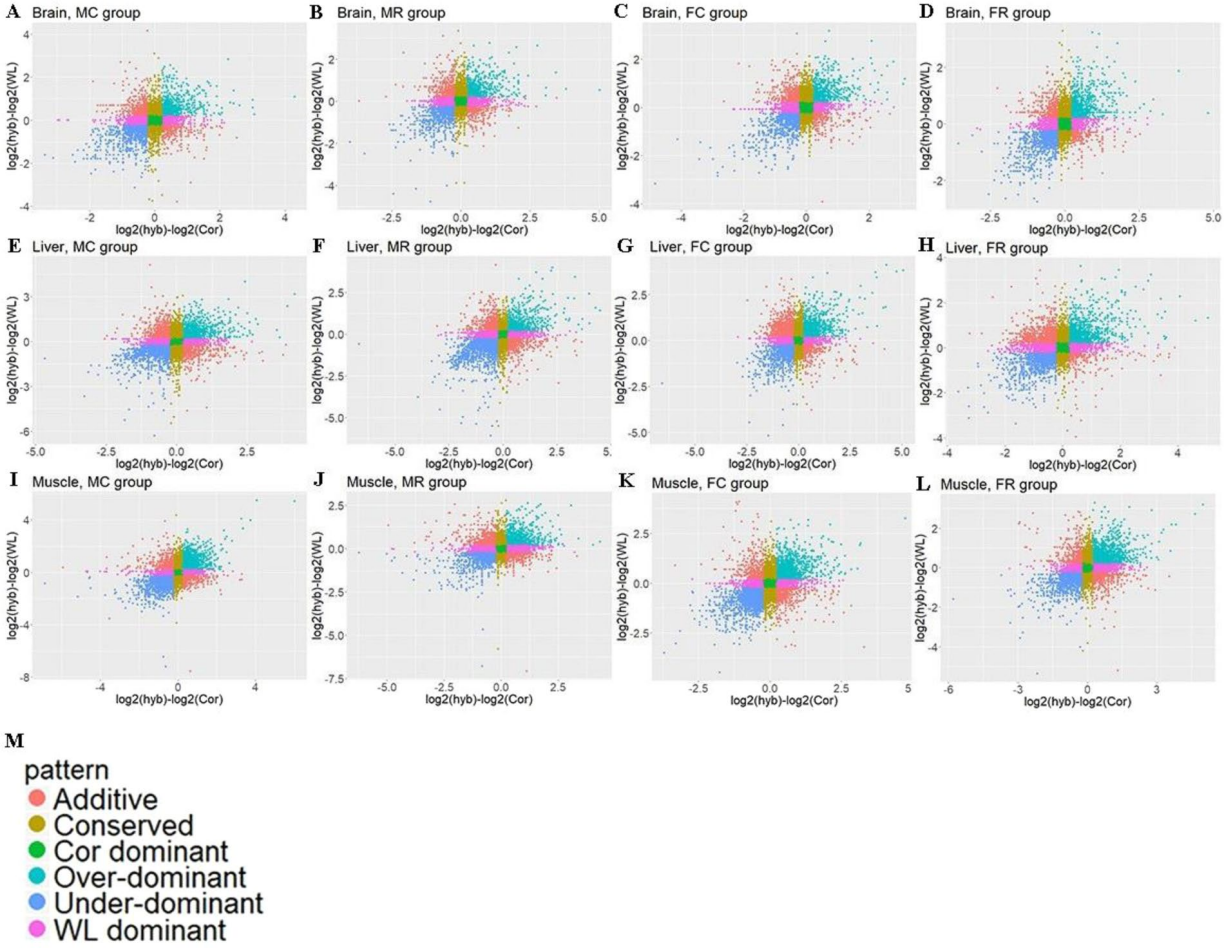
.

**Figure 5:**
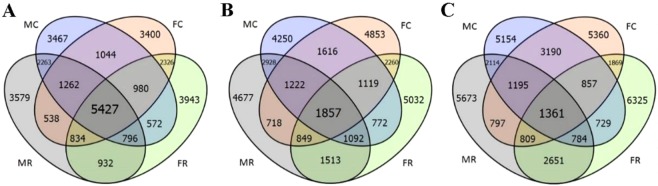
.

**Figure 6:**
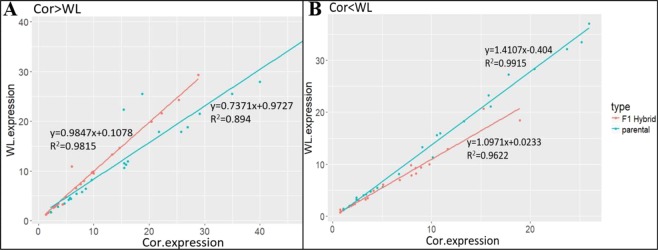
.

**Figure 7:**
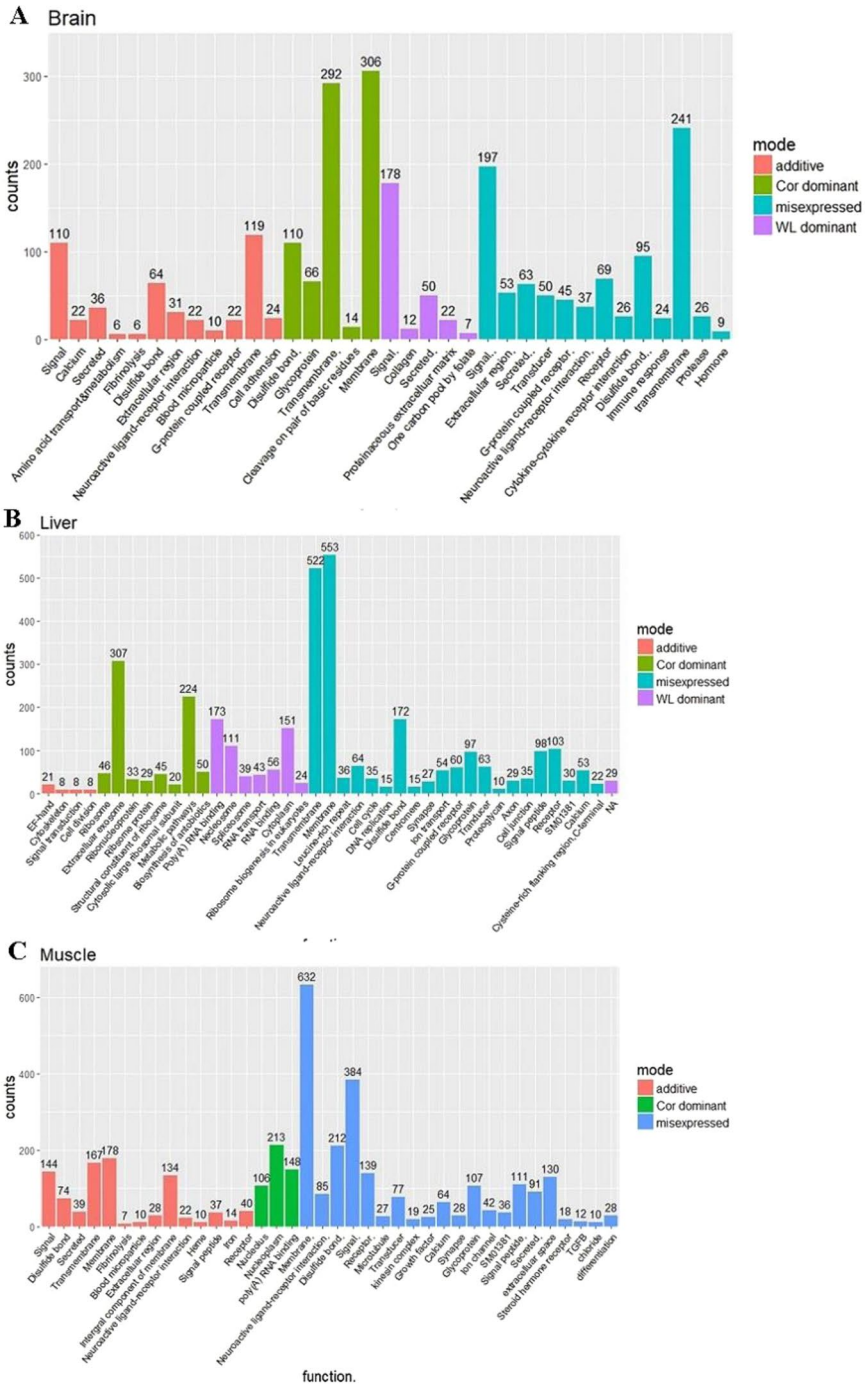
.

