# Supplementary material for: Inheritance patterns of the transcriptome in hybrid chickens and their parents revealed by expression analysis
Source: Sci Rep. 2019 Apr 8;9:5750. doi: 10.1038/s41598-019-42019-x (PMC6453914; doi:10.1038/s41598-019-42019-x)
Supplement: Supplementary file 1 — Supplementary Information [file 41598_2019_42019_MOESM1_ESM.doc]

Inheritance patterns of the transcriptome in hybrid chickens and their parents revealed by expression analysis

Hongchang Gu^†,1^, Xin Qi^†,1^, Yaxiong Jia^2^, Zebin Zhang^3^, Changsheng Nie^1^, Xinghua Li^1^, Junying Li^1^, Zhihua Jiang^4^, Qiong Wang^*,5^& Lujiang Qu^*,1^

^1^Department of Animal Genetics and Breeding, National Engineering Laboratory for Animal Breeding, College of Animal Science and Technology, China Agricultural University, Beijing, China. ^2^Institute of Animal Science, Chinese Academy of Agricultural Sciences, Beijing, China. ^3^Division of Population Genetics, Department of Zoology, Stockholm University, Stockholm, Sweden. ^4^Department of Animal Sciences, Center for Reproductive Biology, Veterinary and Biomedical Research Building, Washington State University, Pullman, United States. ^5^Key Laboratory of Sustainable Development of Marine Fisheries, Ministry of Agriculture, Yellow Sea Fisheries Research Institute, Chinese Academy of Fishery Sciences, Qingdao, China. Hongchang Gu and Xin Qi contributed equally to this work. Correspondence and requests for materials should be addressed to L.Q. (email: quluj@163.com) and Q.W. (email: wangqiong@ysfri.ac.cn).

**Supplementary Table S1.** **Expression of alleles of parents and hybrids in the dominant inheritance pattern.** We used the sum of the normalised values in each pattern to represent allele expression of Cor or WL in parents and offspring. P-value in table refers to correlation of log2(Cor/WL) in hybrids compared with the parental log2(Cor/WL), which was analysed using the Pearson correlation test.

| **tissue** | **pattern** | **group** | **Parents** | | | **Hybrids** | | |  |
| --- | --- | --- | --- | --- | --- | --- | --- | --- | --- |
|  |  |  | Cor | WL | log2(C/W) | Cor | WL | log2(C/W) | |
| **brain** | Cor dominant (Cor>WL) | MC | 5.865 | 4.387 | 0.290 | 3.342 | 3.170 | 0.053 | |
|  |  | FC | 5.679 | 4.541 | 0.224 | 3.592 | 3.446 | 0.041 | |
|  |  | MR | 4.675 | 3.436 | 0.308 | 2.722 | 2.559 | 0.062 | |
|  |  | FR | 9.595 | 8.167 | 0.161 | 4.965 | 4.738 | 0.047 | |
|  | Cor dominant (Cor<WL) | MC | 2.370 | 3.228 | -0.309 | 1.640 | 1.795 | -0.091 | |
|  |  | FC | 3.362 | 4.226 | -0.229 | 2.010 | 2.123 | -0.055 | |
|  |  | MR | 2.445 | 3.119 | -0.243 | 1.060 | 1.162 | -0.092 | |
|  |  | FR | 6.691 | 8.074 | -0.188 | 5.105 | 6.055 | -0.171 | |
|  | WL dominant (Cor>WL) | MC | 2.271 | 1.645 | 0.323 | 1.405 | 1.284 | 0.090 | |
|  |  | FC | 2.179 | 1.736 | 0.227 | 1.324 | 1.209 | 0.091 | |
|  |  | MR | 5.455 | 4.216 | 0.258 | 2.923 | 2.828 | 0.033 | |
|  |  | FR | 3.547 | 2.794 | 0.239 | 1.740 | 1.591 | 0.090 | |
|  | WL dominant (Cor<WL) | MC | 1.061 | 1.387 | -0.267 | 0.838 | 0.924 | -0.097 | |
|  |  | FC | 1.057 | 1.279 | -0.191 | 0.725 | 0.724 | 0.001 | |
|  |  | MR | 3.115 | 4.011 | -0.253 | 2.240 | 2.327 | -0.038 | |
|  |  | FR | 1.668 | 1.990 | -0.176 | 2.231 | 2.354 | -0.053 | |
| **liver** | Cor dominant (Cor>WL) | MC | 39.939 | 27.949 | 0.357 | 28.872 | 29.286 | -0.014 | |
|  |  | FC | 34.944 | 25.535 | 0.314 | 25.350 | 24.303 | 0.042 | |
|  |  | MR | 25.768 | 17.848 | 0.367 | 6.799 | 6.509 | 0.044 | |
|  |  | FR | 18.771 | 25.538 | -0.308 | 13.347 | 13.341 | 0.000 | |
|  | Cor dominant (Cor<WL) | MC | 10.545 | 15.565 | -0.389 | 7.957 | 7.806 | 0.019 | |
|  |  | FC | 9.047 | 13.241 | -0.381 | 8.418 | 8.215 | 0.024 | |
|  |  | MR | 15.981 | 21.095 | -0.278 | 6.789 | 6.942 | -0.022 | |
|  |  | FR | 2.395 | 3.558 | -0.396 | 1.825 | 1.982 | -0.083 | |
|  | WL dominant (Cor>WL) | MC | 15.818 | 11.234 | 0.342 | 9.999 | 9.513 | 0.050 | |
|  |  | FC | 16.154 | 11.870 | 0.308 | 9.850 | 9.828 | 0.002 | |
|  |  | MR | 15.428 | 22.334 | -0.370 | 14.656 | 14.649 | 0.000 | |
|  |  | FR | 15.441 | 10.610 | 0.375 | 9.647 | 9.699 | -0.005 | |
|  | WL dominant (Cor<WL) | MC | 13.288 | 18.252 | -0.317 | 4.116 | 4.916 | -0.178 | |
|  |  | FC | 17.776 | 27.199 | -0.425 | 7.918 | 9.783 | -0.212 | |
|  |  | MR | 15.779 | 23.236 | -0.387 | 8.367 | 9.279 | -0.103 | |
|  |  | FR | 10.887 | 15.904 | -0.379 | 11.619 | 12.944 | -0.108 | |
| **muscle** | Cor dominant (Cor>WL) | MC | 29.071 | 21.498 | 0.302 | 20.448 | 19.930 | 0.026 | |
|  |  | FC | 51.256 | 39.194 | 0.268 | 22.210 | 21.624 | 0.027 | |
|  |  | MR | 7.909 | 5.756 | 0.318 | 4.360 | 3.282 | 0.284 | |
|  |  | FR | 21.738 | 17.856 | 0.197 | 8.243 | 7.996 | 0.030 | |
|  | Cor dominant (Cor<WL) | MC | 20.384 | 28.347 | -0.330 | 9.773 | 9.946 | -0.018 | |
|  |  | FC | 23.686 | 32.133 | -0.305 | 8.939 | 9.316 | -0.041 | |
|  |  | MR | 10.124 | 11.347 | -0.114 | 0.726 | 1.216 | -0.516 | |
|  |  | FR | 3.834 | 4.678 | -0.199 | 3.544 | 3.512 | 0.009 | |
|  | WL dominant (Cor>WL) | MC | 6.908 | 5.427 | 0.241 | 2.865 | 2.704 | 0.058 | |
|  |  | FC | 8.536 | 6.454 | 0.280 | 3.011 | 2.871 | 0.048 | |
|  |  | MR | 26.925 | 18.830 | 0.358 | 6.057 | 10.884 | -0.586 | |
|  |  | FR | 15.505 | 11.492 | 0.299 | 7.604 | 7.384 | 0.029 | |
|  | WL dominant (Cor<WL) | MC | 5.030 | 5.627 | -0.112 | 2.608 | 2.997 | -0.139 | |
|  |  | FC | 4.495 | 5.425 | -0.188 | 3.317 | 3.207 | 0.034 | |
|  |  | MR | 25.937 | 37.031 | -0.356 | 15.248 | 20.612 | -0.301 | |
|  |  | FR | 25.154 | 33.445 | -0.285 | 18.944 | 18.467 | 0.026 | |
| **P-Value** | | 0.0005851 | | | | | | |  |
